# Editorial: The physiological and molecular response of aquatic animals to environmental stresses

**DOI:** 10.3389/fphys.2022.987004

**Published:** 2022-08-25

**Authors:** Wei Xu, Lingling Wang

**Affiliations:** ^1^ Texas A&M University - Corpus Christi, Corpus Christi, TX, United States; ^2^ College of Fisheries and Life Science, Dalian Ocean University, Dalian, China

**Keywords:** aquatic organism, environmental stress, temperature, hypoxia, salinity, ocean acidification (OA), pollutants (environmental), pathogens

The aquatic organisms include approximately 20% of species on the Earth. Many of those aquatic species play essential roles in ecosystems and/or the economy. In the past two hundred years, the significantly increased anthropogenic activities and climate change generated much more pressure on the aquatic organisms. Many species demonstrated phenotypic and genotypic changes in response to environmental pressure. In this Research Topic, “*The physiological and molecular responses of aquatic animals to various environmental stressors*” were discussed.

With the significant global climate change, the aquatic environment starts to become unstable. The most common concern is the temperature, which is directly caused by global warming in the past two centuries. The direct effect of increasing temperature in the aquatic environment on aquatic animals is the change of gene expression profiles upon the thermal stress. With the well-developed, low-cost next-generation sequencing techniques, alterations in transcriptomes under the pressure of high temperature have been identified in many aquatic organisms, including Pacific oyster (*Crassostrea gigas*) (Tan et al.), ark shells (*Scapharca subcrenata*) (Zou et al.), and Farrer’s scallop (*Chlamys farreri*) (Liu et al.). Long-term thermal stress on oysters even resulted in a global divergence in the genome (Tan et al.).

Hypoxia has been long recognized as an environmental stressor that negatively impacts aquatic animals. Since oxygen is critical to oxidation and metabolic activities in animals, lack of oxygen in water showed a significant influence on the post-translational regulation in aquatic animals, such as protein phosphorylation (Sokolov et al.) and gluconeogenesis (Jiang et al.). These changes indicated the activation of alternative metabolic pathways with less oxygen consumption in the animals. The hypoxic stress also impacts the aquatic animals at the transcriptomic level leading to the upregulation of a classic molecular chaperone family, heat shock proteins (Sun et al.), which is similar to that of the animals under thermal stress. Interestingly, hypoxia also influences the brain function in teleost fish (Atlantic croaker, *Micropogonias undulatus*) by manipulating the activity of neuronal nitric oxide synthase (Rahman and Thomas), which explains the behavioral change of the fish associated with hypoxic stress.

If thermal stress and hypoxic stress also affect terrestrial animals, salinity and acidification are two unique stressors to aquatic animals, especially marine species. Changes in marine water salinities are often caused by freshwater input and natural disasters, such as hurricanes and severe storms. The salinity changes usually lead to osmotic stress in marine animals. Compared to the marine vertebrate species, the invertebrate organisms in saltwater are more sensitive to the fluctuations in environmental salinities. A large number of proteins were found to be induced by high or low salinity stress from an Ascidian (*Ciona robusta*). Those proteins are primarily involved in extracellular matrix construction and energy generation (Li et al.). This may explain why the oxygen consumption, ammonia excretion, and calcification in another marine organism (*Creseis acicula*) were affected by salinity stress (Han et al.). It is interesting to see the pathways identified in response to low and high salinity stress were different (Li et al.).

Compared to the salinity change, the formation of ocean acidification is more complicated. The increased CO_2_ level in the atmosphere is the main reason for global ocean acidification while eutrophication is the main contributor to the acidified environment in the coastal areas. Because ocean acidification is a slow process, direct tests on the marine animals in the field before and after the acidification are not realistic. A laboratory setup with increased CO_2_ input to the seawater is the most popular strategy for this type of study. However, this setup can only simulate the acute effect of ocean acidification on animals. Nevertheless, with the increased input of CO_2_, pathways of oxidization and ATP synthesis appeared to be heavily influenced, which indicated that ocean acidification may negatively impact the metabolism of aquatic animals by increasing the cost of energy (Guo et al.).

The aquatic organisms would be considered lucky if they only have to face climate change-related environmental stress. With the increasing agricultural and industrial activities, aquatic pollutants have become greater threats to aquatic animals. Cadmium (Cd), an extremely toxic heavy metal pollutant, had drawn the attention of scientists for decades. The bioaccumulation of Cd often starts from the top of food chains and persistently accumulates in different animals. The toxic effects of Cd in aquatic animals were assessed using a marine species, mantis shrimp (*Oratosquilla oratoria*), as a model. The metabolomic analysis demonstrated oxidative stress and energy metabolism disorders in the host were induced by Cd at 0.05 mg/L concentration (Xu et al.). Besides the heavy metals, the pollution of plastics is another concern to aquatic animal health. As a type of plastic product or degraded product of large plastic waste, microplastics are easier to be accumulated in aquatic organisms, especially the species with small sizes. Fed with polyethylene fibers, the larval Japanese Medaka fish (*Oryzias latipes*) did not show significant problems in gastrointestinal integrity and overall health conditions. However, the polyethylene fibers significantly altered the abundance of Xanthobacteraceae and *Hyphomicrobium* in the gastrointestinal tract of the fish, which may indicate that long-term exposure may result in a chronic issue in larval development (DiBona et al.). So, what will happen to the aquatic animals if both heavy metals and microplastics are present in the water. The answer is it will make aquatic life more miserable. When the zebrafish (*Danio rerio*) were incubated with both microplastics and copper nanoparticles, the hemostasis of the circulatory system was induced (Gao et al.). This may also suggest a potential health risk in humans when exposed to microplastics and copper.

Of course, there are more environmental stressors. Bacteria, such as *Vibrio fluvialis* and *Aeromonas hydrophila*, are reported to be lethal pathogens to fish (Ali et al.; Gewaily et al.). The virulence of *V. fluvialis* in the European seabass (*Dicentrarchus labrax*) was clearly increased with the increasing temperatures (Ali et al.). Although there are very limited measures that can be done to release the stress from climate change and pollutants, the bacterial stress is often relieved by the application of antibiotics, which generates another concern of inducing antibiotic resistance in the environmental bacteria. Fortunately, this concern has been well recognized. One of the alternative strategies was reported. Adding a probiotic bacterium, *Lactobacillus plantarum*, to the diet considerably enhanced the immune system of Nile tilapia (*Oreochromis niloticus*) and consequently made the fish more resistant to *Aeromonas hydrophila* infections (Gewaily et al.).

This is not the end of the story yet. Polycyclic aromatic hydrocarbons (Gan et al.), algal toxins (Kong et al.), desiccation (Chen et al.), and even improper diving behavior (Zhang et al.), were all reported to add more stress to the aquatic organisms. You will probably be wondering how the animals survive with the pressure from the environment.

Recognizing the environmental stress on aquatic animals is the first step. How can the stress be reduced from the living habitats of the animals and the aquaculture environments should be the focus of the next step, which is beyond laboratory research. Measures in research, management, regulation, and perhaps the combination of all these activities are expected to provide a healthy living environment for aquatic organisms in the future.

